# An Unusual Histology for a Lung Nodule: A Case Report of Primary Pulmonary Paraganglioma

**DOI:** 10.3389/fsurg.2021.688236

**Published:** 2021-06-18

**Authors:** Alessandra Mazzucco, Eleonora Poirè, Andrea Leporati, Matteo Chiari, Laura Moneghini, Giorgio Ghilardi, Alessandro Baisi

**Affiliations:** ^1^Thoracic Surgery Unit, University of Milan—Aziende Socio Sanitarie Territoriali (ASST) Santi Paolo e Carlo, Milan, Italy; ^2^Unit of Anatomical Pathology—Aziende Socio Sanitarie Territoriali (ASST) Santi Paolo e Carlo, Milan, Italy

**Keywords:** paraganglioma, thoracic surgery, VATS, lung nodule, rare tumor, case report

## Abstract

**Introduction:** Primary pulmonary paraganglioma is a rare tumor with few cases reported in literature and unspecific clinical presentation.

**Case Presentation:** A 49-year-old woman presented to our department with an incidental finding of a pulmonary mass at chest X-ray and no associated clinical symptom. The CT scan and the FDG-PET showed mild uptake of contrast, but a definitive diagnosis was only possible after surgery through histopathological examination.

**Conclusion:** Paragangliomas originating in the pulmonary tissue are generally non-functioning masses discovered incidentally in otherwise asymptomatic patients. Surgery appears to be the best treatment option, with only radiologic follow-up necessary afterwards.

## Introduction

Primary pulmonary paragangliomas are rare neuroendocrine tumors with few cases reported in the literature. Generally, affected patients show no symptoms and usually discover the mass incidentally during unrelated medical examinations. We report a case of primary pulmonary paraganglioma in an asymptomatic 49-year-old woman.

## Case Presentation

**Figure 1:**
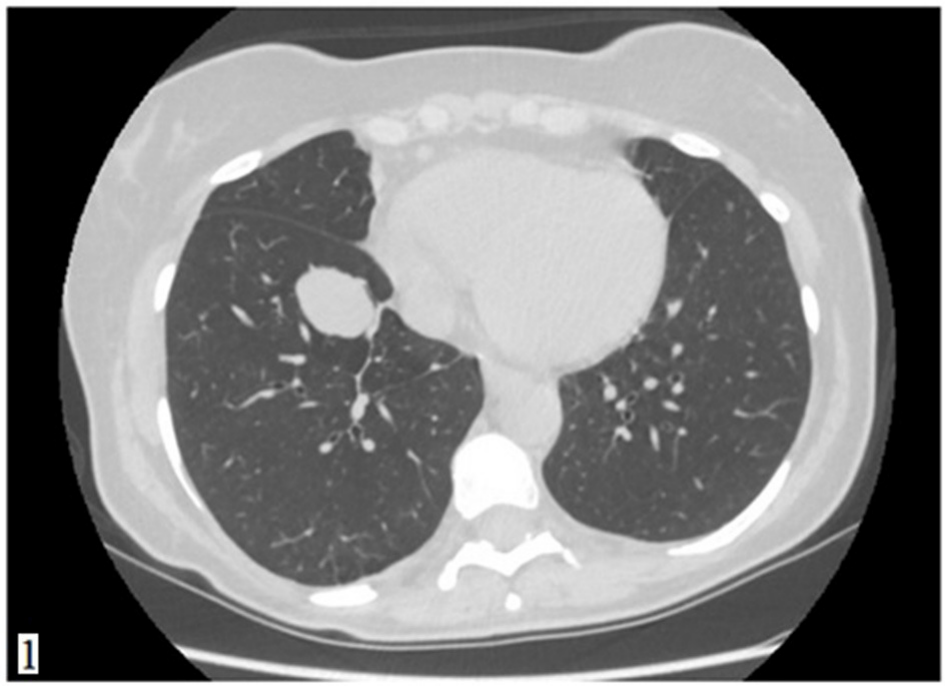
A CT scan showing a solid lesion with well-defined margins in the anterior segment of the inferior lobe of the right lung.

**Figure 2:**
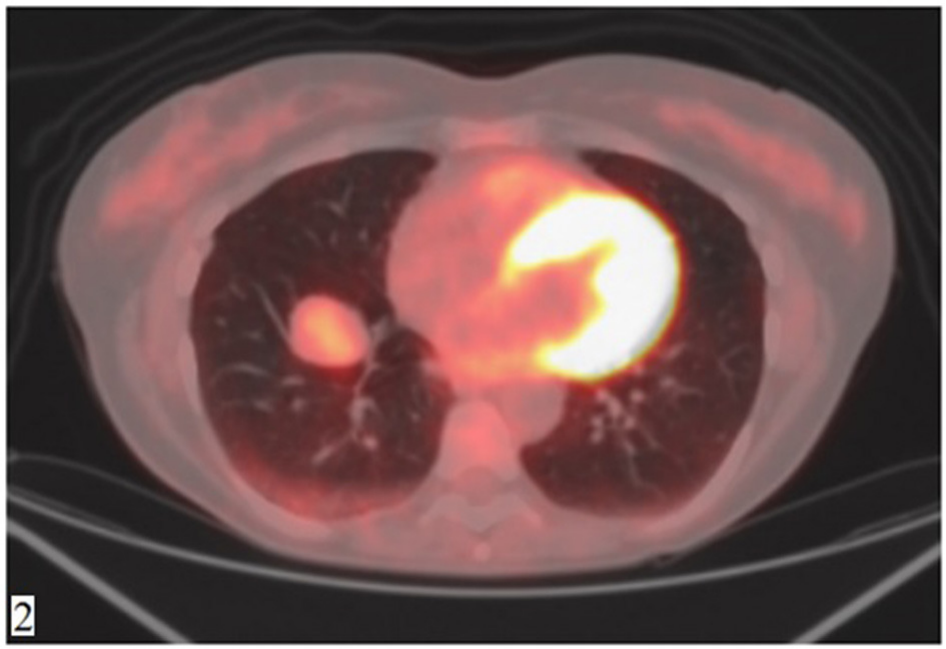
FDG-PET showing mild contrast uptake at the level of the nodule (SUV max 2.8).

**Figure 3:**
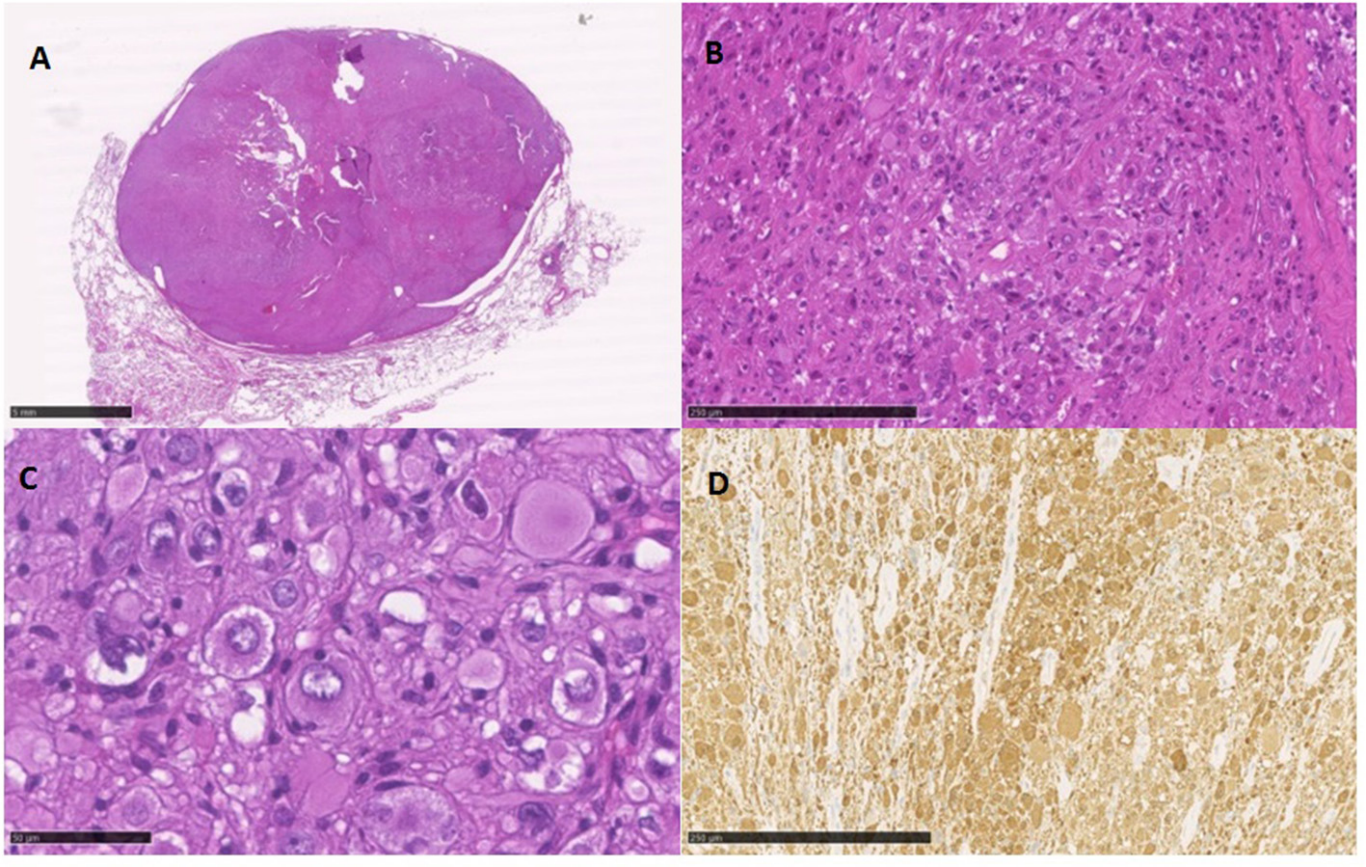
**(A)** low magnification view of the solid, well-defined intrapulmonary neoformation; **(B)** cellular growth is limited by the surrounding fibrous septae; **(C)** the epitheliomorphic ganglion elements are clearly evident with the interposition of some sub-tentacular cells and lymphocytes; **(D)** largely positive immunocytochemical staining for neuron-specific enolase.

A 49-year-old Caucasian woman, an active smoker (15 packs/year) with an otherwise silent past medical history, presented with a dry cough that worsened in the supine position. While the cough resolved with proton-pump inhibitor (PPI) therapy, suggesting a gastrointestinal nature of the symptom, the patient also underwent a routine chest radiograph. The imaging showed a nodule with a diameter of 3.5 cm in the right lower lobe. Thus, a chest CT scan was performed, which confirmed the presence of a solid lesion with well-defined margins, mild contrast enhancement, and a diameter of 34 × 26 mm in the anterior basal segment of the inferior right pulmonary lobe. The exam also revealed an enlarged axillary lymph node that was later confirmed to be inflammatory in nature. To further characterize the lesion, a PET-CT scan with an injection of 18F-fluorodeoxyglucose (FDG) was done; the images showed mild uptake at the level of the nodule in the right lower lobe (with a maximum SUV of 2.8) (Figures 1, 2), suggesting a possibly benign or locally invasive biological behavior. Fine-needle biopsy for typing of the lesion was attempted but ultimately not performed due to poor compliance of the patient during the CT-guided procedure. Therefore, wedge resection of the right inferior lobe and nodal sampling were performed with video-assisted thoracoscopic surgery. A frozen section procedure was performed during the operation, which gave unconclusive results. Hence, it was decided not to perform a completion lobectomy but to wait for the final histological results. The tissue specimen analyzed at histology showed richly vascularized intrapulmonary solid proliferation (Figure 3A) that comprised two types of cells: epitheliomorphic and spindle-shaped cells. The former type of cells had large eosinophilic cytoplasm and moderately atypical nuclei (nucleolates) partially dispersed in a loose stroma crossed by ill-defined septa (Figure 3B); the latter cells, also called “sub-tentacular cells,” were interposed with moderate infiltrate of lympho-plasma cells (Figure 3C). No necrosis was found in the sections examined, and the mitotic index was <1 mitosis for 10 high-magnification fields (10 HPF, 40X). At immunocytochemistry, all cellular elements showed a strong positive reaction for synaptophysin (Syn) and neuron-specific enolase (Figure 3D), while there were only areas of positivity to the S-100 protein in correspondence of the sub-tentacular elements. The dissected lymph nodes were negative. The final diagnosis was a primary pulmonary paraganglioma. The postoperative course was uneventful, with the thoracic drainage removed on the third postoperative day (POD) and no signs of pneumothorax on the chest radiograph performed afterward. The patient was discharged from the hospital on the fourth POD. At the latest checkup, 1 month after the hospital discharge, she showed no sign of relapse on the chest radiograph. After multidisciplinary discussion with the Oncology, Radiology, and Pneumology Departments, it was decided to proceed with radiologic follow-up at 3-month intervals for the first semester with a chest CT scan, then in 6 months for the following year, and later maintain a 1-year radiologic follow-up either with chest radiographs or a CT scan. The main limitation on deciding the timing of the follow-up was the scarcity of available literature on both the treatment and the recurrence rates; however, the available reports seem to suggest an indolent nature of this tumor with an unlikely tendency to recur, which is what informed about our decision on not performing completion of a lobectomy.

## Discussion

Paragangliomas are rare neuroendocrine tumors that arise from the chromaffin cells of the extra-adrenal autonomic paraganglia (1). Their incidence is largely unknown, as they tend to be described in the literature mostly in association with pheochromocytomas; however, the incidence of these tumors combined has been estimated to be about two to eight cases/million a year (2, 3).

Most paragangliomas appear to be sporadic (4), with the majority of patients affected being females and middle-aged (5). Paragangliomas can be associated with either sympathetic or parasympathetic cells; the former are usually “functioning tumors,” as they secrete catecholamines, while the latter have a tendency to be “non-functioning.” Catecholamine-secreting paragangliomas often present with symptoms similar to those of pheochromocytomas (1) (i.e., diaphoresis, headache, and hypertension), whereas non-functioning tumors are silent and are discovered incidentally.

Extra-adrenal paragangliomas are more commonly silent, and if they do present symptoms, these are mostly related to the mass effect at the site of the tumor. Primary pulmonary paragangliomas are rare even among the extra-adrenal paragangliomas, with <30 cases reported in literature (5). The majority of reported patients were asymptomatic and had a nodule discovered during imaging studies of the chest (6). In a minority of cases, there were symptoms consistent with the localization of the mass, such as cough or chest pain (5).

The differential diagnosis of primary pulmonary paraganglioma should include many different conditions, including pulmonary carcinoma, pulmonary tuberculosis, pulmonary mycosis, either round or organizing pneumonia, inflammatory pseudo-tumors, and metastatic lung cancer (5).

Paragangliomas are best evaluated at a contrast-enhanced CT scan, where they show enhancement in the arterial phase (7). However, poor enhancement at a CT scan does not necessarily exclude the diagnosis, but it may be suggestive of a more benign lesion, as it has been reported that the degree of vascularization in non-functioning paragangliomas is not always as rich (5). Silent paragangliomas tend to show mild FDG uptake at a PET scan (5, 7, 8).

These tumors are slow growing and usually benign; however, because they do have a tendency for expansive growth and there are cases reported of low-grade malignant lesions (5, 9), surgical excision, when possible, is the preferred treatment.

In regard to the primary pulmonary paraganglioma, patients have mostly been treated surgically, with either local excision or a lobectomy, depending on the extension of the lesion. No recurrence or metastatic disease has been reported in any of the known cases even though no chemo or radiotherapy was performed afterwards (5, 7). It appears that only 10% of pheochromocytomas and paragangliomas are malignant (4), but there are no biochemical or histological examinations that can reliably predict the tendency of these tumors, and, therefore, surgery remains the only approach to treatment combined with radiologic follow-up.

## Conclusions

This report presents a case of a primary pulmonary paraganglioma, a rare tumor with only a few cases reported in the literature (5, 7, 8). As the patients are mostly asymptomatic, these tumors are generally discovered incidentally. Surgical excision seems to be the best approach to treatment, followed by radiologic follow-up and no further pharmacological therapy.

## Data Availability Statement

The original contributions presented in the study are included in the article/supplementary material, further inquiries can be directed to the corresponding author/s.

## Ethics Statement

Written informed consent was obtained from the individual(s) for the publication of any potentially identifiable images or data included in this article.

## Author Contributions

All authors participated equally in the case and in the writing of the manuscript.

## Conflict of Interest

The authors declare that the research was conducted in the absence of any commercial or financial relationships that could be construed as a potential conflict of interest.
